# Initiating Research in Indian Country: Lessons From the Strong Heart Study

**DOI:** 10.5888/pcd22.240505

**Published:** 2025-04-10

**Authors:** Barbara V. Howard, Elisa T. Lee, Thomas K. Welty, Richard R. Fabsitz

**Affiliations:** 1 Medstar Health Research Institute, Hyattsville, Maryland; 2 University of Oklahoma Health Sciences Center, Oklahoma City; 3 Indian Health Service, Rockville, South Dakota; 4 Missouri Breaks Industries Research, Inc, Eagle Butte, South Dakota

In the 1960s, publications from the Framingham Heart Study indicated that cardiovascular events could have preventable or treatable risk factors (1). Because the participants in that study were White men and women, the question naturally arose about risk factors in other racial and ethnic groups. Subsequently, the National Heart, Lung, and Blood Institute (NHLBI) funded studies to expand research to include Black, Hispanic, and Native Hawaiian populations. Advisors to the NHLBI noted that American Indian populations had not been included. In 1987, the NHLBI released grants that focused on conducting research to better understand cardiovascular disease in American Indian populations.

NHLBI senior staff were skeptical about this project because of the rural populations and the role of tribal sovereignty. Initial funding provided for a 3-year study in 3 groups of American Indians. Grants were awarded for 3 field centers 1) to recruit and examine a cohort of Northern Plains Indians in the Dakotas and establish a cardiology reading center, 2) to recruit and examine an American Indian cohort from the multiple tribes in southwestern Oklahoma and establish a data coordinating center, and 3) to recruit and examine an American Indian cohort in southwestern Arizona and establish a core laboratory. During the past 35 years, 7 phases of the Strong Heart Study (SHS) have been funded and 2 cohorts have been recruited and examined (2,3). The original cohort focused on adult men and women aged 45 to 74 years (1). The second cohort focused on large families with members aged 15 years or older (2). The objective of this essay is to assist health care advocates who are contemplating, or currently working on, research in Indian Country. Many of the points addressed may be applicable to studies conducted in other remote or isolated populations.

## Challenges in Conducting Research in Indian Country

American Indian tribes are autonomous and vary in community size, approach to governance, cultural norms, English-speaking ability, and economic development. Conduct of a multicenter study must be prepared to deal with these differences and their potential effect on recruitment and scientific translation.

The NHLBI recognized some potential challenges and took actions to address them. For example, to ensure that selected grantees had an existing mutually respectful working relationship with the tribes, preselection site visits were made to potential grantees. These visits required a meeting that included leadership from the participating tribes so that the relationships could be evaluated.

Site visits also explored the challenges in transportation, logistics, facilities, and personnel in conducting clinical examinations at each center. Distances from grantee institutions to study sites and within study sites were unusually long, with sites ranging from no reservations in Oklahoma to reservations spanning more than 4,000 square miles in South Dakota. Transportation options were limited and often affected by severe weather conditions that could dramatically affect research budgets and timelines.

Examination facilities were limited and often required the cooperation of the Indian Health Service (IHS) and local tribes. Early support from the IHS director facilitated access to examination rooms and medical records. Tribes often provided meeting space for steering committee and community meetings. Hiring and training local personnel to recruit and examine study participants turned out to be win–win: we found dedicated excellent staff from the communities who could be trained for the study needs, and who, in turn, trained the outside staff on cultural norms, ways of life, and approaches to day-to-day challenges. A detailed study manual facilitated initial training, periodic retraining, and constant referencing. All interviewers and clinical staff were centrally trained in examination procedures and certified by qualified experts. Quality control measures were built into the examinations and study procedures.

Clinic space and examination equipment often were not available and had to be acquired or transported to the multiple examination sites at the study centers. Centers relied on mobile vans that were designed and equipped to support examinations. Finally, timely transport of samples was often constrained due to distances between examination sites and shipping facilities. As a result, the study protocol required modification to allow samples to be properly stored and periodically shipped to the central laboratory.

## Key Areas and Important Steps in Conducting Research

Based on the SHS experience, we summarize key areas and important steps to conduct a research project in American Indian communities (Table).

**Table:** Important Considerations When Establishing and Implementing a Research Project in Diverse Communities: Reflections From the Strong Heart Study

Key area	Important consideration
Building trust	Meet with community leaders and community members before proposal is submitted and after funding
	Explain goals of the project and describe the importance in addressing needs in the community
	Describe the potential benefits to the individual participants and to the community
	Describe in detail the planned methods, including any potential impact or risks

Transparent implementation	Emphasize that community members will be employed for all possible roles
	Describe the procedures and how the members will be trained to perform them
	Explain the meaning of quality control procedures

Sharing results and mutual feedback	Describe how results will be provided to participants and their health care providers and also available to communities for funding opportunities
	Ask for suggestions at each step and implement as many as possible
	Provide a summary report to the community leaders at the end of the project to bring study closure but also promote continuing partnership

### Building trust

Establishing and maintaining trust and good working relationships with the communities is the most important step to conduct a research project in an American Indian community (4). Holding study meetings in the participating communities whenever possible builds awareness of the study within the community, promotes the relationship between study staff and study participants, and allows study staff to better understand the culture, opportunities, and challenges at each study site. Tribal leadership is often overburdened with the business of the tribe. The governance processes for approvals by the tribes are likely to vary. Working patiently and cooperatively with the tribe not only built support for the SHS but also promoted a long-term partnership providing mutual benefit.

SHS investigators communicated with tribal leaders, tribal health boards, and communities before, during, and after funding to inform them of the opportunity to participate in the study, to obtain approvals in every area where examinations would be performed, and to share what was learned. The tribes are most interested in what they can expect to gain from participating in the study. It is essential for investigators to align their interests with the tribes’ interests. Taking every opportunity to explain the goals and methods of the project, what is to be gained, what are the risks, and how the community will benefit contributes to a successful project outcome.

### Transparent conduct of the study

Hiring from within the community, when possible, is very important. Having staff who understand how to navigate challenges and address difficult situations is useful, and training staff from the community means that they become advocates for the research study. In the SHS, we used NHLBI training programs to hire and train people from high school students to postgraduates. The success of that approach is evident from the long list of students who have chosen to pursue medical careers or assume key roles in this and other research projects in Indian Country (4). Requirements, such as quality control procedures, are often considered a waste of time by community staff and participants and must be explained as a critical part of any research endeavor. Increasing understanding will increase community acceptance of the study procedures. One example is the storage and use of samples for future research that could not necessarily be envisioned at the time of the initial study. After discussions with communities, stored samples and data were blessed by a spiritual leader in a “cedaring” ceremony and provision was made for the return or proper disposal of unused samples at study’s end. The value of stored samples is easily illustrated in a recent study (5) in which urine samples were used to address exposure to heavy metals that have now been linked to the risk of cardiovascular disease.

### Sharing results and mutual feedback

Sharing individual and study results is essential for a respectful and lasting partnership with the study community. Ideally, dialogue with the community improves the process (6,7). A summary of examination findings is provided to each participant. Scientific manuscripts and abstracts are shared with the tribes before presentation. A periodic newsletter for participants, tribes, and others has been published since 1989. A website describing all aspects of the SHS is available online (https://strongheartstudy.org). In addition, educational brochures are published on various subjects (eg, diabetes, high blood pressure, diet) for participants, and tribes are assisted in preparing their reports and proposals for funding. For example, tribes were assisted in applying for a diabetes project funded by the IHS. In 2022, a successful program (Strongheart Tribal Approach to Research) to fund tribal research was established to provide funds for tribal members to initiate and conduct research on their own interests. This program provided research experience and built awareness and appreciation for the process of conducting studies in the community.

Finally, investigators must also be willing to listen to community feedback about the conduct of the study, including what is missing, ineffective, or unacceptable, and what the priorities of the community are. In SHS, we found that the communities were very interested in the next generation. They wanted their children to be included in the study so the children would have healthier lives. As a result, the investigators proposed a family study that opened new avenues for research, such as research on risk factors in the young, disease trends, generational differences, genetics, and other “omics” studies. SHS has demonstrated that listening and working closely across populations that have unique risk factor profiles, lifestyles, and environmental exposures can help investigators gain clinical insights and develop public health approaches to improving population health (4,5,7,8). SHS continues to progress with new explorations of environmental factors, social determinants of health, cognitive function, and additional omics. SHS has demonstrated that research conducted thoughtfully, respectfully, and in cooperation with the populations being studied offers substantial scientific opportunities.
